# Prevalence of Osteoporosis and Fracture in China: The China Osteoporosis Prevalence Study

**DOI:** 10.1001/jamanetworkopen.2021.21106

**Published:** 2021-08-16

**Authors:** Linhong Wang, Wei Yu, Xiangjun Yin, Lijia Cui, Shunyu Tang, Ning Jiang, Lu Cui, Nan Zhao, Qiang Lin, Lin Chen, Hua Lin, Xiaolan Jin, Zhong Dong, Zeping Ren, Zhulin Hou, Yongqing Zhang, Jieming Zhong, Shunxiang Cai, Yuan Liu, Ruilin Meng, Ying Deng, Xianbin Ding, Jingang Ma, Zhongjian Xie, Lin Shen, Wen Wu, Mengmeng Zhang, Qifeng Ying, Yuhong Zeng, Jin Dong, Steven R. Cummings, Zhixin Li, Weibo Xia

**Affiliations:** 1 National Center for Chronic and Non-communicable Disease Control and Prevention, Chinese Center for Disease Control and Prevention, Beijing, China; 2 Department of Radiology, Peking Union Medical College Hospital, Chinese Academy of Medical Sciences and Peking Union Medical College, Beijing, China; 3 Division of Elderly Health, National Center for Chronic and Noncommunicable Disease Control and Prevention, Chinese Center for Disease Control and Prevention, Beijing, China; 4 Department of Endocrinology, Key Laboratory of Endocrinology, National Commission of Health, Peking Union Medical College Hospital, Chinese Academy of Medical Sciences and Peking Union Medical College, Beijing, China; 5 Medical Sciences Research Center, Peking Union Medical College Hospital, Chinese Academy of Medical Sciences and Peking Union Medical College, Beijing, China; 6 Department of Wound Repair and Rehabilitation Medicine, State Key Laboratory of Trauma, Burns and Combined Injury, Daping Hospital, Army Medical University, Chongqing, China; 7 Department of Orthopaedics, Nanjing Drum Tower Hospital, the Affiliated Hospital of Nanjing University Medical School, Nanjing, Jiangsu Province, China; 8 Department of Endocrinology, Chengdu Military General Hospital, Chengdu, Sichuan Province, China; 9 Beijing Center for Diseases Prevention and Control, Beijing, China; 10 Shanxi Provincial Center for Disease Control and Prevention, Taiyuan, Shanxi Province, China; 11 Jilin Provincial Center for Disease Control and Prevention, Changchun, Jilin Province, China; 12 Jiangsu Provincial Center for Disease Control and Prevention, Nanjing, Jiangsu Province, China; 13 Zhejiang Provincial Center for Disease Control and Prevention, Hangzhou, Zhejiang Province, China; 14 Hubei Provincial Center for Disease Control and Prevention, Wuhan, Hubei Province, China; 15 Hunan Provincial Center for Disease Control and Prevention, Changsha, Hunan Province, China; 16 Guangdong Provincial Center for Disease Control and Prevention, Guangzhou, Guangdong Province, China; 17 Sichuan Center for Disease Control and Prevention, Chengdu, Sichuan Province, China; 18 Chongqing Center for Disease Control and Prevention, Chongqing, China; 19 Shaanxi Provincial Center for Disease Control and Prevention, Xi'an, Shaanxi Province, China; 20 Hunan Provincial Key Laboratory of Metabolic Bone Diseases, National Clinical Research Center for Metabolic Diseases, Department of Metabolism and Endocrinology, The Second Xiangya Hospital of Central South University, Changsha, Hunan Province, China; 21 Department of Integrated Traditional Chinese and Western Medicine, Union Hospital, Tongji Medical College, Huazhong University of Science and Technology, Wuhan, Hubei Province, China; 22 Department of Endocrinology, Guangdong Provincial People’s Hospital, Guangdong Academy of Medical Sciences, Guangdong Provincial Geriatrics Institute, Guangzhou, Guangdong Province, China; 23 Department of Osteoporosis, Jilin FAW General Hospital, Changchun, Jilin Province, China; 24 Center of Osteoporosis, Zhejiang Provincial People's Hospital, Hangzhou, Zhejiang Province, China; 25 Department of Osteoporosis, Honghui Hospital, Xi’an Jiaotong University, Xi’an, Shaanxi Province, China; 26 Department of Endocrinology, The First Affiliated Hospital of Shanxi Medical University, Taiyuan, Shanxi, China; 27 San Francisco Coordinating Center, California Pacific Medical Center Research Institute, San Francisco, California

## Abstract

**Importance:**

The aging of the population is associated with an increasing burden of fractures worldwide. However, the epidemiological features of fractures in mainland China are not well known.

**Objective:**

To assess the prevalence of and factors associated with osteoporosis, clinical fractures, and vertebral fractures in an adult population 40 years or older in mainland China.

**Design, Setting. and Participants:**

This cross-sectional study, the China Osteoporosis Prevalence Study, was conducted from December 2017 to August 2018. A random sample of individuals aged 20 years or older who represented urban and rural areas of China were enrolled, with a 99% participation rate.

**Main Outcomes and Measures:**

Weighted prevalence of osteoporosis, clinical fracture, and vertebral fracture by age, sex, and urban vs rural residence as determined by x-ray absorptiometry, questionnaire, and radiography.

**Results:**

A total of 20 416 participants were included in this study; 20 164 (98.8%; 11 443 women [56.7%]; mean [SD] age, 53 [13] years) had a qualified x-ray absorptiometry image and completed the questionnaire, and 8423 of 8800 (95.7%) had a qualified spine radiograph. The prevalence of osteoporosis among those aged 40 years or older was 5.0% (95% CI, 4.2%-5.8%) among men and 20.6% (95% CI, 19.3%-22.0%) among women. The prevalence of vertebral fracture was 10.5% (95% CI, 9.0%-12.0%) among men and 9.7% (95% CI, 8.2%-11.1%) among women. The prevalence of clinical fracture in the past 5 years was 4.1% (95% CI, 3.3%-4.9%) among men and 4.2% (95% CI, 3.6%-4.7%) among women. Among men and women, 0.3% (95% CI, 0.0%-0.7%) and 1.4% (95% CI, 0.8%-2.0%), respectively, with osteoporosis diagnosed on the basis of bone mineral density or with fracture were receiving antiosteoporosis treatment to prevent fracture.

**Conclusions and Relevance:**

In this cross-sectional study of an adult population in mainland China, the prevalence of osteoporosis and vertebral fracture were high and the prevalence of vertebral fracture and clinical fracture was similarly high in men and women. These findings suggest that current guidelines for screening and treatment of fractures among patients in China should focus equally on men and women and should emphasize the prevention of vertebral fractures.

## Key Points

**Question:**

What is the prevalence of osteoporosis and clinical and vertebral fracture in the adult population of mainland China?

**Findings:**

In this cross-sectional study of 20 416 individuals, the prevalence of osteoporosis among adults 40 years or older was 5.0% among men and 20.6% among women, and the prevalence of vertebral fracture was 10.5% among men and 9.7% among women.

**Meaning:**

These findings suggest that recommendations for screening and treatment of fractures should include both men and women in China.

## Teaser

This cross-sectional study evaluates the prevalence of and factors associated with osteoporosis, clinical fractures, and vertebral fractures among adults 40 years or older in mainland China by sex, age group, and urban vs rural residence.

## Introduction

Fractures are associated with a substantial burden on health systems that increases with the increasing number of older adults.^1^ The population of China constitutes one-fifth of the world’s population and an even greater proportion of the older population.^2^ The prevalence of fractures in the whole population in China is not known, but few people in China receive drug treatment to prevent fracture.^3^ For example, it was reported that only 6.5% of people in China received medications for treatment of osteoporosis within 6 months after a fracture.^4^

A previous study^5^ showed that vertebral fractures are common in women in Beijing, China. Defining fractures on the basis of the reduction of vertebral heights on lateral radiographs of the thoracic and lumbar spine,^6^ a study reported that the prevalence of vertebral fractures among women in Beijing ranged from 13% among those aged 50 to 59 years to more than 50% among women older than 80 years.^5^ However, to our knowledge, there have been no nationwide community-based studies on the epidemiological characteristics of osteoporosis and osteoporotic fracture in China that have included men and women in urban and rural areas.^3^

Therefore, we undertook the China Osteoporosis Prevalence Study, a national population-based screening project of men and women aged 20 years or older from representative regions and urban and rural areas in China to assess the prevalence of osteoporosis, clinical fractures, and vertebral fractures in a population in mainland China. We assessed bone mineral density (BMD) and fractures as well as factors associated with low BMD and fracture.

## Methods

### Study Design and Participants

This cross-sectional study was conducted in China from December 2017 to August 2018. The study protocol was approved by the ethical review committee of the Chinese Center for Disease Control and Prevention. We obtained written informed consent from all study participants, and confidentiality of information was assured. The study followed the Strengthening the Reporting of Observational Studies in Epidemiology (STROBE) reporting guideline.

**Figure 1.**
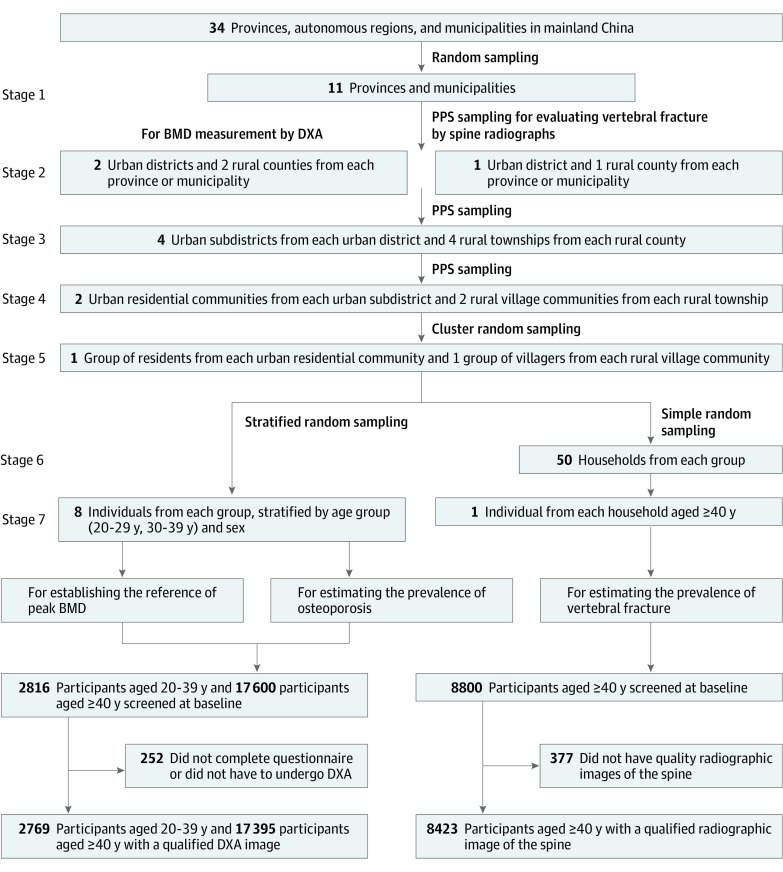
Flowchart of Participant Selection A multistage stratified cluster random sampling method was used in the study to enroll a sample of people who would be representative of adults in China. Details are given in the Study Design and Participants subsection of the Methods section. BMD indicates bone mineral density; DXA, dual x-ray absorptiometry; and PPS, probability proportionate to size.

The China Osteoporosis Prevalence Study enrolled a representative sample of 20 416 participants aged 20 years or older from mainland China. We used a multistage stratified cluster random sampling method to enroll a sample of people who would be representative of adults in China (Figure 1 and eTable 1, eTable 2, and eFigure 1 in the Supplement). In stage 1, we randomly selected 11 of 34 provinces and municipalities. In stage 2, we used probability-proportional-to-size sampling (PPS) to randomly select 2 urban districts and 2 rural counties from each province or municipality in which to perform dual x-ray absorptiometry (DXA) and to randomly select 1 urban district and 1 rural county in which to perform spine radiography. In stage 3, we used PPS to randomly select 4 urban subdistricts from each urban district or 4 townships from each rural county. In stage 4, we used PPS to randomly select 2 urban residential communities from each urban subdistrict or 2 rural village communities from each township. In stage 5, we used a cluster random sampling method to select 1 group of residents from each urban residential community or 1 group of villagers from each rural village community. In stages 6 and 7, we randomly selected 8 participants aged 20 to 39 years from each group of residents or villagers stratified by age group (20-29 years and 30-39 years) and sex to establish the reference of peak BMD in individuals in China; we selected 50 participants aged 40 years or older from 50 households in each group of residents or villagers to estimate the prevalence of osteoporosis. The process of sample size calculation is given in the eMethods in the Supplement. Only inaccessible households were replaced. The alternative household should have had demographic structures similar to those of the original households and should have been from the same sample group. The replacement rate was less than 10%. The inclusion and exclusion criteria of participants are shown in the eMethods in the Supplement.

### Data Collection

Trained interviewers administered a standardized questionnaire including information for demographic characteristics, medical history, and risk factors during in-person interviews. The definition of risk factors in the questionnaire is shown in the eMethods in the Supplement.

We transported all participants from the same urban district or rural county to a local hospital where a DXA scanner was available or to a mobile vehicle with a DXA scanner if DXA scanners were not available locally. Certified technicians performed DXA for each participant to measure BMD in the lumbar spine (L1 to L4), femoral neck, and total hip using Hologic scanners (Hologic Inc) or GE-Lunar scanners (GE Healthcare). Protocols of cross-calibration among different DXA scanners are given in the eMethods in the Supplement.

Trained technicians performed lateral radiography of thoracic and lumbar spine for 8800 participants from a randomly selected urban district and county in each province or municipality. We centered a beam at T7 for thoracic spine (covering the level T4 to L1) and L2 for lumbar spine (covering the level T12 to L5). Two skeletal radiologists (W.Y. and Q.L.) assessed vertebral fracture independently based on a semiquantitative technique of Genant et al,^6^ and fractures of grade 1 or higher were included. When there was a disagreement, the image was reassessed to reach a consensus.

### Statistical Analysis

The aim of the China Osteoporosis Prevalence Study was to describe the prevalence of osteoporosis based on BMD, vertebral fracture, and clinical fracture (defined as fracture events recalled by participants on a questionnaire) in the past 5 years among men and women in 5 age groups (40-49 years, 50-59 years, 60-69 years, 70-79 years, and ≥80 years) and in urban and rural areas. We calculated sample weights by sampling clusters and poststratification weights based on the 2010 National Census of China,^7^ and we calculated the final weights as sample weights multiplied by poststratification weights to represent the general population in China.

We established a mean BMD and SD database for men and women and plotted lumbar spine, femoral neck, and total hip BMD by age and sex using Svysmooth, a smoothing procedure available in the survey package of R, version 4.0.0 (R Project for Statistical Computing).^8^ We diagnosed osteoporosis based on the peak BMD (SD) in young men and women aged 20 to 39 years that was established in the present study. We used the diagnostic criteria of the World Health Organization: T scores = (BMD − peak BMD of individuals of the same sex)/(SD of peak BMD of individuals of the same sex).^9^ We defined individuals with T scores of −2.5 or less in any sites (L1 to L4, femoral neck, or total hip) as having osteoporosis. We calculated the weighted prevalence of osteoporosis by sex, age group, and urban vs rural setting. We calculated weighted prevalence of vertebral fracture (based on radiographic findings as described above) and clinical fracture in the past 5 years (based on a questionnaire) by sex, age group, and urban vs rural settings. We calculated the treatment rate among patients with T scores of −2.5 or less in any sites or with a history of fracture (vertebral fracture of grade 2 or higher based on radiographic findings or clinical fracture in the past 5 years based on a questionnaire) and current use of antiosteoporosis treatment (including bisphosphonate, calcitonin, estrogen, parathyroid hormone analogue, selective estrogen receptor modulator, or an active form of vitamin D or its analogue).

We performed multivariable linear regression to investigate factors associated with BMD in the lumbar spine, femoral neck, and total hip. We performed multivariable logistic regression to investigate factors associated with vertebral fracture of grade 2 or higher and clinical fracture in the past 5 years. We used data from all participants for whom the variables of interest were available for analysis and did not impute missing data. We adjusted all *P* values for multiple testing, present 95% CIs, and considered a 2-sided *P* < .05 as statistically significant. We performed all data analysis using SAS, version 9.3 (SAS Institute Inc).

## Results

A total of 20 416 participants were included in this study and were enrolled for DXA, including 2816 participants aged 20 to 39 years and 17 600 participants aged 40 years or older; 8800 participants aged 40 years or older were enrolled for spine radiography. Of those identified and invited to participate in the study, 20 164 (98.8%; 11 443 women [56.7%]; mean [SD] age, 53 [13] years) had qualified DXA images and 8423 (95.7%) had qualified spine radiographs. The participation rate was 99%. The demographic characteristics of participants are presented in eTables 1 and 2 in the Supplement.

The peak mean (SD) BMDs at the lumbar spine (L1 to L4), femoral neck, and total hip were 1.00 (0.12) g/cm^2^, 0.87 (1.41) g/cm^2^, and 0.92 (0.14) g/cm^2^, respectively, for men and 1.04 (0.13) g/cm^2^, 0.82 (0.13) g/cm^2^, and 0.89 (0.13) g/cm^2^, respectively, for women (eTable 3 and eFigure 2 in the Supplement). The peak BMD in men was reached at 20 to 29 years of age at all measured sites, and the peak BMD in women was reached at 30 to 39 years of age in the lumbar spine and 20 to 29 years of age in the femoral neck and total hip. The overall prevalence of osteoporosis was 20.6% (95% CI, 19.3%-22.0%) among women aged 40 years or older and 5.0% (95% CI, 4.2%-5.8%) among men 40 years or older (Table 1 and eTable 4 in the Supplement). The prevalence of osteoporosis among postmenopausal women was 32.1% (95% CI, 30.1%-34.1%), and the prevalence of osteoporosis among men 50 years or older was 6.9% (95% CI, 5.8%-8.0%). The prevalence of osteoporosis was similar in urban areas (4.6%; 95% CI, 3.3%-5.9%) and rural areas (5.3%; 95% CI, 4.2%-6.3%) (*P* = .43) among men and higher in rural areas (22.3%; 95% CI, 20.5%-24.3%) than in urban areas (17.3%; 95% CI, 15.8%-18.8%) (*P* < .001) among women.

**Table 1.** Weighted Prevalence of Osteoporosis in the Population 40 Years or Older in China by Age, Sex, and Residence Type

Characteristic	Prevalence, % (95% CI)	
	Men (n = 7150)	Women (n = 9826)
Age, y		
40-49	2.4 (1.3-3.5)	4.3 (2.4-6.1)
50-59^a^	4.6 (3.3-5.8)	16.1 (14.2-18.0)
60-69^a^	5.4 (4.1-6.7)	37.1 (34.5-39.7)
70-79^a^	12.3 (9.0-15.5)	51.3 (46.4-56.2)
≥80^a^	21.9 (9.7-34.1)	67.5 (56.5-78.4)
≥40^a^	5.0 (4.2-5.8)	20.6 (19.3-22.0)
*P* value for trend	<.001	<.001
Residence type		
Urban^a^	4.6 (3.3-5.9)	17.3 (15.8-18.8)
Rural^a^	5.3 (4.2-6.3)	22.3 (20.5-24.3)
*P* value for difference	.43	<.001

^a^ *P* < .05 for men vs women.

**Figure 2.**
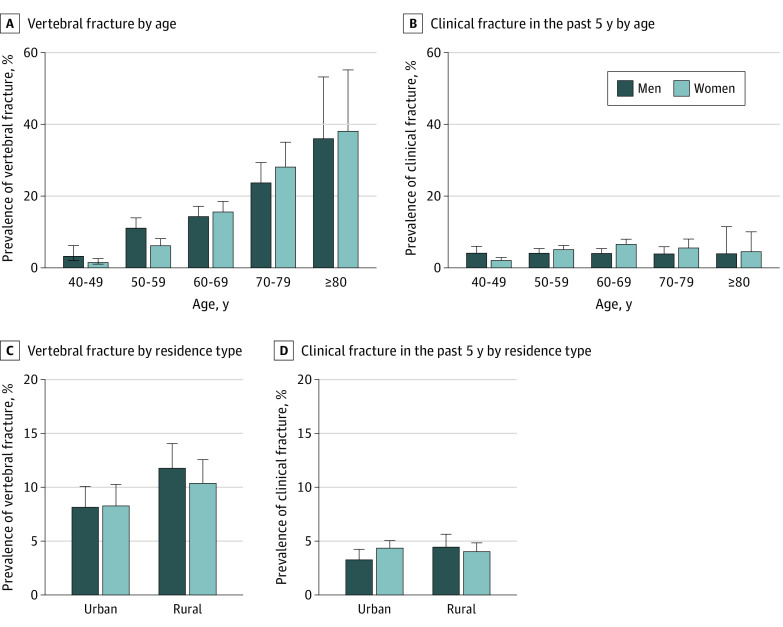
Prevalence of Fracture in the Population Aged 40 Years or Older in China Vertebral fracture was defined on the basis of findings of radiography of the thoracic and lumbar spine. Clinical fracture in the past 5 years was defined on the basis of questionnaire response.

Thoracic and lumbar spine radiographs were obtained for 8800 participants, and 377 were judged as unqualified and were thus excluded. The prevalence of vertebral fracture, defined on the basis of radiographic findings, increased with age and was similar among men and women (Table 2 and Figure 2). Among individuals aged 40 years or older, the prevalence of vertebral fracture was 10.5% (95% CI, 9.0%-12.0%) among men and 9.7% (95% CI, 8.2%-11.1%) among women. When only vertebral fractures of grade 2 or higher were included, the prevalence of vertebral fracture was 3.8% (95% CI, 2.7%-4.9%) among men and 4.8% (95% CI, 3.7%-5.9%) among women (Table 2). The prevalence of clinical fracture in the past 5 years was 4.1% (95% CI, 3.3%-4.9%) among men and 4.2% (95% CI, 3.6%-4.7%) among women, which was lower than the prevalence of vertebral fracture. The prevalence of vertebral fracture was significantly higher among men in rural areas (11.8%; 95% CI, 9.7%-13.9%) than in urban areas (8.2%; 95% CI, 6.6%-9.9%) (*P* = .008) but was similar among women in rural (10.4%; 95% CI, 8.5%-12.4%) and urban (8.3%; 95% CI, 6.5%-10.0%) areas (*P* = .98). The prevalence of clinical fracture in the past 5 years was also similar among men in urban (3.3%; 95% CI, 2.4%-4.2%) and rural (4.5%; 95% CI, 3.4%-5.6%) areas (*P* = .09) and among women in urban (4.4%; 95% CI, 3.7%-5.1%) and rural (4.1%; 95% CI, 3.3%-4.8%) areas (*P* = .58). Despite the high prevalence of osteoporosis and fracture, only 0.3% (95% CI, 0.0%-0.7%) of men and 1.4% (95% CI, 0.8%-2.0%) of women with osteoporosis based on BMD or with fracture were receiving antiosteoporosis treatment to prevent fracture (eTable 5 in the Supplement).

**Table 2.** Weighted Prevalence of Vertebral Fracture and Clinical Fracture in the Population in China by Age, Sex, and Residence Type

Characteristic	Prevalence, % (95% CI)					
	Vertebral fracture^a^		Vertebral fracture of grade 2 or higher^a^		Clinical fracture in past 5 y^b^	
	Men (n = 3589)	Women (n = 4834)	Men (n = 3589)	Women (n = 4834)	Men (n = 7384)	Women (n = 10 082)
Age, y						
40-49	3.2 (1.0-5.4)	1.5 (0.8-2.3)	1.4 (0.0-3.4)	0.4 (0.0-0.8)	4.2 (2.6-5.7)^c^	2.1 (1.4-2.8)
50-59	11.1 (8.5-13.7)^c^	6.2 (4.5-8.0)	3.1 (1.7-4.4)	2.4 (1.3-3.5)	4.1 (2.9-5.3)	5.1 (4.0-6.2)
60-69	14.3 (11.6-16.9)	15.5 (12.7-18.2)	5.2 (3.6-6.8)	6.9 (4.9-9.0)	4.1 (3.0-5.2)	6.6 (5.3-7.8)
70-79	23.8 (18.7-29.0)	28.1 (21.7-34.5)	9.1 (5.6-12.6)^c^	16.5 (11.0-22.0)	3.9 (2.3-5.5)	5.6 (3.6-7.6)
≥80	36.0 (20.1-51.8)	38.1 (22.1-54.1)	17.2 (3.5-30.8)	22.4 (8.2-36.7)	4.0 (0.0-8.3)	4.5 (0.8-8.2)
≥40	10.5 (9.0-12.0)	9.7 (8.2-11.1)	3.8 (2.7-4.9)	4.8 (3.7-5.9)	4.1 (3.3-4.9)^c^	4.2 (3.6-4.7)
*P* value for trend	<.001	<.001	<.001	<.001	>.99	<.001
Residence type						
Urban	8.2 (6.6-9.9)	8.3 (6.5-10.0)	2.4 (1.6-3.3)	4.1 (2.6-5.6)	3.3 (2.4-4.2)^c^	4.4 (3.7-5.1)
Rural	11.8 (9.7-13.9)	10.4 (8.5-12.4)	4.6 (3.0-6.2)	5.1 (3.6-6.6)	4.5 (3.4-5.6)^c^	4.1 (3.3-4.8)
*P* value for difference	.008	.98	.02	.36	.09	.58

^a^ Vertebral fracture was defined by 2 independent radiologists on the basis of findings on lateral radiography of the thoracic and lumbar spine.

^b^ Clinical fracture was defined as fracture events recalled by participants on a questionnaire.

^c^ *P* < .05 for men vs women.

In the multivariable linear regression analysis (eTable 6 in the Supplement), the following factors were significantly associated with lower BMD in the lumbar spine (L1 to L4): female sex (β, −70.1; 95% CI, −80.3 to −57.7), older age (50-59 years: β, −65.5 [95% CI, −74.1 to −56.9]; 60-69 years: β, −101.5 [95% CI, −110.8 to −92.2]; 70-79 years: β, −125.7 [95% CI, −139.7 to −111.7]; ≥80 years, β, −159.6 [95% CI, −188.6 to −147.4]; *P* < .001 for trend), lower body mass index (β, −99.3; 95% CI, −121.5 to −77.1), ever smoker (β, −16.9; 95% CI, −28.7 to −5.1), positive Sharpened Romberg test result (β, −21.0, 95% CI, −31.6 to −10.3), and rural residence (β, −22.6; 95% CI, −29.7 to −15.5). The multivariable logistic regression model identified several characteristics associated with fracture in adults aged 40 years or older (Table 3). A vertebral fracture of grade 2 or higher was associated with male sex (odds ratio [OR], 1.82; 95% CI, 1.07-3.30), older age (50-59 years: OR, 1.81 [95% CI, 0.58-5.64]; 60-69 years: OR, 2.91 [95% CI, 0.91-9.36]; 70-79 years: OR, 4.84 [95% CI, 1.42-16.50]; ≥80 years: OR, 5.21 [95% CI, 1.30-20.98]; *P* < .001 for trend), lower femoral neck BMD (OR, 2.35; 95% CI, 1.83-3.01), and a positive Sharpened Romberg test result (OR, 1.59; 95% CI, 1.08-2.35). In addition, risk of clinical fracture in the past 5 years was greater among individuals who had lower femoral neck BMD (OR, 1.35; 95% CI, 1.15-1.58), had overweight (OR, 1.31; 95% CI, 1.02-1.68), consumed alcohol (OR, 1.64; 95% CI, 1.04-2.60), used glucocorticoids for more than 3 months (OR, 3.06; 95% CI, 1.20-7.83), and had a longer sit-to-stand completion time (7.2-8.9 s: OR, 1.16 [95% CI, 0.79-1.71]; 9.0-10.8 s: OR, 1.36 [95% CI, 0.92-2.00]; >10.8 s: OR, 1.89 [95% CI, 1.26-2.83]; *P* = .01 for trend). Sex-specific regression models for BMD and fracture are shown in eTables 7 to 10 in the Supplement.

**Table 3.** Multivariable Analysis of Factors Associated With Vertebral Fracture of Grade 2 or Higher and Clinical Fracture in the Population 40 Years or Older in China

Variable	Vertebral fracture of grade 2 or higher (n = 8357)		Clinical fracture in the past 5 y (n = 17 299)	
	Adjusted OR (95% CI)^a^	*P* value	Adjusted OR (95% CI)^a^	*P* value
Female	0.55 (0.30-0.93)	.03	1.09 (0.75-1.58)	.63
Age, y				
40-49	1 [Reference]	NA	1 [Reference]	NA
50-59	1.81 (0.58-5.64)	.31	1.17 (0.81-1.68)	.38
60-69	2.91 (0.91-9.36)	.07	1.10 (0.73-1.64)	.63
70-79	4.84 (1.42-16.50)	.01	0.82 (0.50-1.34)	.44
≥80	5.21 (1.30-20.98)	.02	0.66 (0.28-1.57)	.35
*P* value for trend	<.001	NA	.25	NA
Femoral neck BMD, by SD decrement	2.35 (1.83-3.01)	<.001	1.35 (1.15-1.58)	<.001
Body mass index^b^				
<18.5	0.33 (0.14-0.76)	.009	0.54 (0.26-1.13)	.10
18.5-23.9	1 [Reference]	NA	1 [Reference]	NA
≥24	1.19 (0.80-1.77)	.39	1.31 (1.02-1.68)	.03
*P* value for trend	.75	NA	.02	NA
Parent fractured hip	0.47 (0.22-1.00)	.05	1.24 (0.82-1.88)	.29
Ever smoked	0.53 (0.26-1.04)	.07	1.09 (0.67-1.77)	.70
Consumed alcohol	1.75 (0.79-3.84)	.17	1.64 (1.04-2.60)	.03
Glucocorticoid use for >3 mo	1.28 (0.44-3.76)	.65	3.06 (1.20-7.83)	.02
Gait speed, m/s				
<0.70	1.60 (0.76-3.36)	.21	1.09 (0.76-1.54)	.62
0.70-0.84	1.79 (0.83-3.86)	.14	1.11 (0.76-1.62)	.57
0.85-1.01	1.69 (0.83-3.44)	.15	1.03 (0.72-1.47)	.85
>1.01	1 [Reference]	NA	1 [Reference]	NA
*P* value for trend	.22	NA	.85	NA
5-Repetition sit-to-stand test completion time, s				
<7.2	1 [Reference]	NA	1 [Reference]	NA
7.2-8.9	2.39 (1.19-4.79)	.01	1.16 (0.79-1.71)	.43
9.0-10.8	2.15 (1.02-4.53)	.04	1.36 (0.92-2.00)	.11
>10.8	2.67 (1.18-6.05)	.02	1.89 (1.26-2.83)	.002
*P* value for trend	.13	NA	.01	NA
Positive Sharpened Romberg test result	1.59 (1.08-2.35)	.02	1.18 (0.89-1.57)	.22
Rural residence	1.22 (0.83-1.80)	.32	1.10 (0.87-1.38)	.39

Abbreviations: BMD, bone mineral density; NA, not applicable; OR, odds ratio.

^a^ Adjusted for sex, age, femoral neck BMD, body mass index, parent fractured hip, ever smoked, consumed alcohol, glucocorticoid use for more than 3 months, gait speed, and 5-repetition sit-to-stand test completion time.

^b^ Calculated as weight in kilograms divided by height in meters squared.

## Discussion

To our knowledge, this cross-sectional study is the largest nationwide, population-based study of BMD, the prevalence of osteoporosis, and the prevalence of fracture in the population of mainland China. Previous studies on the prevalence of osteoporosis and vertebral fracture in China were regional studies with small sample size^5,10,11^ or enrolled only urban citizens^12^ or individuals who were undergoing a physical examination.^13,14^ The participation rate of the present study was 99%. Thus, the difference among regions and other possible confounding factors was minimized and analysis was possible across different subgroups. We found a high prevalence of osteoporosis and fracture in China; 5.0% of men and 20.6% of women aged 40 years or older had osteoporosis, and 10.5% of men and 9.7% of women aged 40 years or older had vertebral fracture. The prevalence of vertebral fracture was similar among men and women. This finding suggests that prevention of vertebral fractures in China should focus on both men and women. In addition, we found that osteoporosis and fracture remained undertreated; Only 0.3% of men and 1.4% of women with osteoporosis or a history of fracture had received antiosteoporosis treatment. Having overweight, a positive Sharpened Romberg test result, and a longer sit-to-stand completion time were identified as factors associated with osteoporosis or fracture, suggesting that appropriate weight management and reducing the risk of falls may be associated with a lower burden of fracture in China.

The prevalence of osteoporosis increased in association with increasing age and was significantly higher among women than among men, which was similar to previous findings.^13,15^ The prevalence of osteoporosis among women in China was higher than that among women in most American and European countries^16,17,18,19^ but similar to that among women in other countries in Asia (Korea^20^ and Japan^21^). However, the prevalence of osteoporosis among men in China was similar to that among men in other countries.^16,17,18,19,20,21^ A possible explanation for the high prevalence of osteoporosis among women in Asia is earlier or faster bone loss associated with aging, which may be attributed to genetic factors and lower intake of calcium, vitamin D, and protein in the traditional Asian diet.^22^

Vertebral fracture is a common complication of osteoporosis^3^ and accounted for most fractures (Table 2). Several methods have been developed for assessing vertebral fracture on radiographs.^23^ We used a semiquantitative method in this study to assess vertebral fracture because it is more widely used in epidemiological studies^5,24,25,26^ and thus allowed more consistent comparison of our results with published prevalence data. We included grade 1 fractures in our definition of vertebral fracture, but the associations with age, sex, and other factors were similar for fractures of grade 2 or higher. Compared with previous studies, the prevalence of vertebral fracture was lower than that reported by Cui et al^5^ in 2017 in a study of postmenopausal women in Beijing and higher than that reported by Kwok et al^24^ in 2013 in a study of the population of Hong Kong.

Of interest, the prevalence of vertebral fracture was similar among men and women (Table 2). The difference in prevalence of vertebral facture by sex has varied in different studies.^27,28,29^ A regional study^27^ of only citizens of China found that the prevalence of vertebral fracture among men aged 60 to 70 years was higher than that among women in the same age group but was lower among men than among women in older age groups. A small epidemiological study^28^ in countries in Asia found a higher prevalence of vertebral fracture among men aged 65 years or older than among women aged 65 years or older in Japan, Indonesia, Hong Kong, and Thailand. A microsimulation model established for European countries estimated that the prevalence of vertebral fracture among men younger than 64 years was similar to that among women in the same age group, and in older age groups, the prevalence was lower among men than among women.^29^ The decreased androgen levels and loss of muscle strength associated with aging, increased exposure to farm or construction work, and increased use of vehicles may contribute to the occurrence of fracture in older men.

Female sex, age, and lower body mass index were significantly associated with lower BMD, similar to previous findings.^30^ A positive Sharpened Romberg test result was also associated with lower BMD, suggesting that decreased muscle mass or function may contribute to decreased balance and mobility and may be associated with lower BMD. In accordance with previous studies,^31^ low femoral neck BMD and overweight were associated with vertebral fracture and clinical fracture. We also found that a positive Sharpened Romberg test result and longer sit-to-stand time were associated with vertebral fracture or clinical fracture. These findings support the association of impaired muscle function with increased risk of fall and fracture in the population in China^32,33^ and highlights the need for efforts to reduce falls in the population in China that has a high risk of osteoporosis.

Our study has important clinical and public health implications. We found a high prevalence of osteoporosis and fracture in all age groups nationwide in China. With the aging of the population, the prevalence of osteoporosis and fracture is anticipated to continue to increase in China. Current policies regarding osteoporotic fracture prevention in many countries have mainly focused on postmenopausal women,^34,35^ but our data highlighted the importance of early recognition of high risk of fracture in both men and women based on risk factors and not just BMD. Specifically, promotion of appropriate weight management and reducing the risk of falls should be public health priorities. This study may provide the information for future guideline development and public policy formulation in China.

### Limitations

This study has limitations. First, because this was a cross-sectional study, findings regarding factors associated with osteoporosis and fracture may have been affected by uncertain confounding effects. Prospective longitudinal studies in the population of China may better examine this association. Second, we assessed the patterns of vertebral fracture by analyzing fractures of grade 1 or higher, which might include nonosteoporotic deformities; thus, we also separately analyzed vertebral fractures of grade 2 or higher to achieve more specific findings. Furthermore, we defined clinical fracture based on self-report, which may have a recall bias. A multicenter fracture registry system is being established in China, and future studies will use data on history of fracture collected from this system. In addition, the non-Han population, which has different genetic characteristics and lifestyle, was not included in the study. Ethnicity-specific reference data should be established in future studies.

## Conclusions

In this cross-sectional study, the prevalence of osteoporosis and fracture in China was high. The prevalence of vertebral fracture was similar among men and women. The prevalence of vertebral fracture was higher than that of other clinical fractures in the past 5 years at older ages. Clinical and public health efforts to reduce the burden of fractures in China should be devoted equally to men and women in urban and rural areas and should focus on screening and prevention of vertebral fractures.

## Notes

Supplement. **eTable 1.** Demographic characteristics of the study subjects with qualified DXA **eTable 2.** Demographic characteristics of the study subjects with qualified spine radiographs **eTable 3.** Mean BMDs, SDs of L1-4, femoral neck (FN), and total hip (TH) in male and female (g/cm^2^) **eTable 4.** Weighted prevalence of osteoporosis at various skeletal sites in Chinese population aged 40 years or older, based on peak BMD in the current study **eTable 5.** Percentage of those patients with BMD T≤−2.5 or with fracture who received anti-osteoporosis treatment **eTable 6.** Linear regression analysis of risk factors associated with BMD in the Chinese adult population aged 40 years or older **eTable 7.** Linear regression analysis of risk factors associated with BMD in Chinese men aged 40 years or older **eTable 8.** Linear regression analysis of risk factors associated with BMD in Chinese women aged 40 years or older **eTable 9.** Multiply-adjusted odds ratios for vertebral fracture of grade 2 or above, and clinical fracture associated with risk factors in Chinese men aged 40 years or older **eTable 10.** Multiply-adjusted odds ratios for vertebral fracture of grade 2 or above, and clinical fracture associated with risk factors in Chinese women aged 40 years or older **eFigure 1.** Geographic distribution of 11 selected provinces/municipalities in the study **eFigure 2.** Smoothed curve of mean bone mineral density (BMD) by age in Chinese population aged 20 years or older
eMethods.
