# Supplementary material for: Prevalence of Osteoporosis and Fracture in China: The China Osteoporosis Prevalence Study
Source: JAMA Netw Open. 2021 Aug 16;4(8):e2121106. doi: 10.1001/jamanetworkopen.2021.21106 (PMC8369359; doi:10.1001/jamanetworkopen.2021.21106)
Supplement: Supplement. — eTable 1. Demographic characteristics of the study subjects with qualified DXA eTable 2. Demographic characteristics of the study subjects with qualified spine radiographs eTable 3. Mean BMDs, SDs of L1-4, femoral neck (FN), and total hip (TH) in male and female (g/cm2) eTable 4. Weighted prevalence of osteoporosis at various skeletal sites in Chinese population aged 40 years or older, based on peak BMD in the current study eTable 5. Percentage of those patients with BMD T≤−2.5 or with fracture who received anti-osteoporosis treatment eTable 6. Linear regression analysis of risk factors associated with BMD in the Chinese adult population aged 40 years or older eTable 7. Linear regression analysis of risk factors associated with BMD in Chinese men aged 40 years or older eTable 8. Linear regression analysis of risk factors associated with BMD in Chinese women aged 40 years or older eTable 9. Multiply-adjusted odds ratios for vertebral fracture of grade 2 or above, and clinical fracture associated with risk factors in Chinese men aged 40 years or older eTable 10. Multiply-adjusted odds ratios for vertebral fracture of grade 2 or above, and clinical fracture associated with risk factors in Chinese women aged 40 years or older eFigure 1. Geographic distribution of 11 selected provinces/municipalities in the study eFigure 2. Smoothed curve of mean bone mineral density (BMD) by age in Chinese population aged 20 years or older eMethods. [file jamanetwopen-e2121106-s001.pdf]

Supplemental Online Content

Wang L, Yu W, Yin X, et al. Prevalence of osteoporosis and fracture in China: the China Osteoporosis Prevalence Study. *JAMA Netw Open.* 2021;4(8):e2121106. doi:10.1001/jamanetworkopen.2021.21106

eTable 1. Demographic characteristics of the study subjects with qualified DXA

eTable 2. Demographic characteristics of the study subjects with qualified spine radiographs

eTable 3. Mean BMDs, SDs of L1-4, femoral neck (FN), and total hip (TH) in male and female (g/cm^2)

eTable 4. Weighted prevalence of osteoporosis at various skeletal sites in Chinese population aged 40 years or above, based on peak BMD in the current study

eTable 5. Percentage of those patients with BMD T≤−2.5 or with fracture who received anti-osteoporosis treatment

eTable 6. Linear regression analysis of risk factors associated with BMD in the Chinese adult population aged 40 years or older

eTable 7. Linear regression analysis of risk factors associated with BMD in Chinese men aged 40 years or older

eTable 8. Linear regression analysis of risk factors associated with BMD in Chinese women aged 40 years or older

eTable 9. Multiply-adjusted odds ratios for vertebral fracture of grade 2 or above, and clinical fracture associated with risk factors in Chinese men aged 40 years or older

eTable 10. Multiply-adjusted odds ratios for vertebral fracture of grade 2 or above, and clinical fracture associated with risk factors in Chinese women aged 40 years or older

eFigure 1. Geographic distribution of 11 selected provinces/municipalities in the study

eFigure 2. Smoothed curve of mean bone mineral density (BMD) by age in Chinese population aged 20 years and over

eMethods. The process of sample size calculation in the study.

This supplemental material has been provided by the authors to give readers additional information about their work.

© 2021 Wang L et al. *JAMA Network Open.*

eTable 1. Demographic characteristics of the study subjects with qualified DXA

	Total		Male		Female	
	n	%	n	%	n	%
Total	17395	100	7348	42.2	10047	57.8
Age						
40-49	4619	26.6	1966	26.8	2653	26.4
50-59	5260	30.2	2098	28.6	3162	31.5
60-69	5485	31.5	2323	31.6	3162	31.5
70-79	1756	10.1	825	11.2	931	9.3
≥80	275	1.6	136	1.9	139	1.4
Residence						
Urban	9483	54.5	3748	51.0	5735	57.1
Rural	7912	45.5	3600	49.0	4312	42.9
Area						
North	6360	36.6	2722	37.0	3638	36.2
South	11035	63.4	4626	63.0	6409	63.8
Race						
Han	17247	99.1	7297	99.3	9950	99.0
Minority	147	0.8	51	0.7	96	1.0
Education						
Illiterate/semiliterate	1440	8.3	273	3.7	1167	11.6
Primary school	5162	29.7	2065	28.1	3097	30.8
Junior school	6001	34.5	2816	38.3	3185	31.7
Senior school/Technical school	3117	17.9	1424	19.4	1693	16.9
College degree or above	1672	9.6	769	10.5	903	9.0
Marital status						
Single	120	0.7	94	1.3	26	0.3
Married/cohabiting	15736	90.5	6867	93.5	8869	88.3
Widowed/divorced/separate	1536	8.8	386	5.3	1150	11.4

© 2021 Wang L et al. *JAMA Network Open.*

eTable 2. Demographic characteristics of the study subjects with qualified spine radiographs

	Total		Male		Female	
	**n**	**%**	**n**	**%**	**n**	**%**
Total	8423	100	3589	42.6	4834	57.4
Age						
40-49	2189	26.0	920	25.6	1269	26.3
50-59	2571	30.5	1020	28.4	1551	32.1
60-69	2650	31.5	1155	32.2	1495	30.9
70-79	886	10.5	431	12.0	455	9.4
≥80	127	1.5	63	1.8	64	1.3
Residence						
Urban	4610	54.7	1817	50.6	2793	57.8
Rural	3813	45.3	1772	49.4	2041	42.2
Area						
North	3113	37.0	1323	36.9	1790	37.0
South	5310	63.0	2266	63.1	3044	63.0
Race						
Han	8372	99.4	3569	99.4	4803	99.4
Minority	50	0.6	20	0.6	30	0.6
Education						
Illiterate/semiliterate	643	7.6	120	3.3	523	10.8
Primary school	2585	30.7	1031	28.7	1554	32.1
Junior school	2989	35.5	1414	39.4	1575	32.6
Senior school/Technical school	1463	17.4	679	18.9	784	16.2
College degree or above	741	8.8	345	9.6	396	8.2
Marital status						
Single	60	0.7	47	1.3	13	0.3
Married/cohabiting	7599	90.2	3339	93.0	4260	88.1
Widowed/divorced/separate	762	9.0	203	5.7	559	11.6

© 2021 Wang L et al. *JAMA Network Open.*

eTable 3. Mean BMDs, SDs of L1-4, femoral neck (FN), and total hip (TH) in male and female (g/cm^2)

Site	Age (years)	Male				Female			
		N	Mean	SD	95% CI	N	Mean	SD	95% CI
L1-4	20-29	701	1.00	0.12	(0.99, 1.01)	691	1.02	0.13	(1.01, 1.03)
	30-39	666	1.00	0.14	(0.99, 1.01)	699	1.04	0.13	(1.03, 1.05)
	40-49	1956	0.97	0.14	(0.97, 0.98)	2638	1.00	0.15	(1.00, 1.01)
	50-59	2067	0.95	0.15	(0.95, 0.96)	3111	0.89	0.16	(0.89, 0.90)
	60-69	2259	0.96	0.18	(0.95, 0.97)	3057	0.79	0.15	(0.78, 0.79)
	70-79	780	0.94	0.19	(0.93, 0.95)	879	0.75	0.16	(0.74, 0.76)
	80-	124	0.93	0.21	(0.89, 0.96)	126	0.72	0.15	(0.69, 0.75)
FN	20-29	702	0.87	0.14	(0.86, 0.88)	692	0.82	0.13	(0.82, 0.84)
	30-39	668	0.84	0.13	(0.83, 0.85)	696	0.82	0.13	(0.81, 0.83)
	40-49	1952	0.82	0.13	(0.81, 0.82)	2649	0.79	0.13	(0.79, 0.80)
	50-59	2087	0.78	0.13	(0.78, 0.79)	3152	0.73	0.13	(0.72, 0.73)
	60-69	2315	0.76	0.13	(0.76, 0.77)	3155	0.66	0.12	(0.65, 0.66)
	70-79	818	0.71	0.13	(0.70, 0.72)	925	0.58	0.11	(0.58, 0.59)
	80-	136	0.68	0.14	(0.65, 0.70)	137	0.55	0.13	(0.53, 0.58)
TH	20-29	702	0.92	0.14	(0.91, 0.93)	690	0.89	0.13	(0.88, 0.90)
	30-39	668	0.91	0.14	(0.90, 0.92)	696	0.88	0.12	(0.88, 0.89)
	40-49	1952	0.91	0.14	(0.90, 0.91)	2645	0.88	0.14	(0.87, 0.89)
	50-59	2087	0.88	0.14	(0.88, 0.89)	3148	0.82	0.14	(0.81, 0.82)
	60-69	2315	0.86	0.14	(0.86, 0.87)	3154	0.73	0.12	(0.73, 0.74)
	70-79	818	0.82	0.14	(0.81, 0.83)	924	0.66	0.13	(0.65, 0.66)
	80+	136	0.77	0.14	(0.74, 0.79)	136	0.62	0.14	(0.60, 0.64)

© 2021 Wang L et al. *JAMA Network Open.*

eTable 4. Weighted prevalence of osteoporosis at various skeletal sites in Chinese population aged 40 years or above, based on peak BMD in the current study

	Prevalence of osteoporosis % (95% CI)		
	**L1-L4**	**Femoral neck**	**Total hip**
Age (Years)			
40-49	2.4 (1.8,3.2)	1.0 (0.2,1.9)	1.0 (0.2,1.8)
50-59	8.7 (7.7,9.9)	1.7 (1.2,2.1)	2.2 (1.7,2.7)
60-69	18.8 (17.3,20.4)	4.9 (4.1,5.6)	5.2 (4.3,6)
70-79	26.8 (23.8,30.1)	14.4 (11.8,17.0)	14.2 (11.6,16.7)
80+	38.2 (29.8,47.5)	26.0 (18.9,33.1)	28.4 (20.5,36.4)
40 and over	10.6 (9.9,11.3)	4.1 (3.6,4.7)	4.4 (3.8,4.9)
p for trend	<.001	<.001	<.001

© 2021 Wang L et al. *JAMA Network Open.*

eTable 5. Percentage of those patients with BMD T ≤ -2.5 or with fracture who received anti-osteoporosis treatment[a]

	Men % (95% CI)	Women % (95% CI)	Total % (95% CI)
Age (Years)			
40-49	0	1.2 (0 to 3.7)	0.6 (0 to 1.8)
50-59	0.7 (0 to 2.0)	1.4 (0 to 2.9)	1.2 (0.1 to 2.3)
60-69	0.7 (0 to 1.9)	1.7 (0.9 to 2.4)	1.5 (0.8 to 2.1)
70-79	0	1.8 (0.4 to 3.1)	1.3 (0.3 to 2.3)
≥80	0	0	0
Total (40+)	0.3 (0 to 0.7)	1.4 (0.8 to 2.0)	1.1 (0.6 to 1.5)

[a] The history of anti-osteoporosis treatment was based on the medication history including bisphosphonate, calcitonin, estrogen, parathyroid hormone analogue, selective estrogen receptor modulator and active form of vitamin D or its analogue in the questionnaire; the fracture history included both vertebral fracture of grade 2 or above in the radiographs and clinical fracture in the past five years in the questionnaire.

© 2021 Wang L et al. *JAMA Network Open.*

eTable 6. Linear regression analysis of risk factors associated with BMD in the general Chinese adult population aged 40 years or older

	L1-L4 (N=17357)		Total hip (N=17380)	
	**Regression coefficient (95%CI)**	***p* value**	**Regression coefficient (95%CI)**	***p* value**
Female sex	-70.1 (-80.3, -57.7)	<.001	-72.2 (-80.6,-63.8)	<.001
Age (years)				
40-49	0.0 (ref)		0.0 (ref)	
50-59	-65.5 (-74.1,-56.9)	<.001	-45.6 (-53.1,-38.0)	<.001
60-69	-101.5 (-110.8,-92.2)	<.001	-89.5 (-97.6,-81.4)	<.001
70-79	-125.7 (-139.7,-111.7)	<.001	-141.9 (-153.0,-130.8)	<.001
⩾80	-159.6 (-188.6,-147.4)	<.001	-185.9 (-207.1,-164.6)	<.001
p for trend	<.001		<.001	
Body-mass index (kg/m^2)				
<18·5 (underweight)	-99.3 (-121.5,-77.1)	<.001	-90.5 (-107.4,-73.6)	<.001
18·5–23·9 (normal weight)	0.0 (ref)		0.0 (ref)	
⩾24・0 (overweight and obese)	61.8 (54.4,69.2)	<.001	76.7 (70.5,82.9)	<.001
p for trend	<.001		<.001	
Parent Fractured Hip	-1.7 (-18.5,15.0)	.83	-12.1 (-25.4,1.2)	.08
Ever-smoker	-16.9 (-28.7,-5.1)	.005	-6.3 (-16.0,3.3)	.19
Alcohol consumption	0.6 (-13.1,14.5)	.92	4.4 (-6.5,15.3)	.43
Glucocorticoid use >3 months	-13.1 (-34.1,7.9)	.22	0.6 (-24.2,25.5)	.95
Gait speed (m/s)				
<0.70	4.7 (-5.8,15.4)	.37	-0.3 (-9.0,8.3)	.93
0.70-0.84	6.2 (-4.2,16.6)	.24	6.5 (-2.2,15.3)	.14
0.85-1.01	1.7 (-8.5,12.0)	.74	4.8 (-4.0,13.7)	.28
>1.01	0.0 (ref)		0.0(ref)	
p for trend	.32		.16	
Five times sit to stand test completion time (s)				
<7.2	0.0 (ref)		0.0(ref)	
7.2-8.9	-3.6 (-14.3,7.0)	.50	5.0 (-4.0,14.2)	.27
9.0-10.8	-9.5 (-19.8,0.8)	.07	2.1 (-6.3,10.7)	.61
>10.8	-8.6 (-19.5,2.3)	.12	-3.4 (-12.7,5.8)	.47
p for trend	.31		0.52	
Sharpened Romberg test positive	-21.0 (-31.6,-10.3)	.001	-23.6 (-33.4,-13.7)	<.001
Rural residents	-22.6 (-29.7,-15.5)	<.001	-8.9 (-14.6,-3.1)	.002

© 2021 Wang L et al. *JAMA Network Open.*

eTable 7. Linear regression analysis of risk factors associated with BMD in Chinese men aged 40 years or older

	L1-L4 (N=7335)		Total hip (N=7351)	
	**Regression coefficient (95% CI)**	***p* value**	**Regression coefficient (95% CI)**	***p* value**
Age				
40-49	0.0 (ref)		0.0 (ref)	
50-59	-16.1 (-28.4,-3.9)	.01	-23.1 (-33.9,-12.2)	<.001
60-69	1.4 (-11.6,14.6)	.82	-36.6 (-48.0,-25.2)	<.001
70-79	-14.5 (-34.0,4.9)	.14	-73.9 (-89.5,-58.3)	<.001
≥80	-40.5 (-86.0,4.8)	.08	-123.5 (-151.0,-95.9)	<.001
p for trend	.01		<.001	
Body-mass index (kg/m^2)				
<18·5 (underweight)	-99.8 (-127.5,-72.2)	<.001	-82.6 (-107.0,-58.1)	<.001
18·5–23·9 (normal weight)	0.0 (ref)		0.0 (ref)	
≥24·0 (overweight and obese)	67.5 (56.8,78.2)	<.001	77.8 (68.9,86.8)	<.001
p for trend	<.001		<.001	
Parent Fractured Hip	5.8 (-18.6,30.3)	.63	-7.0 (-27.1,13.1)	.49
Ever-smoker	-22.6 (-34.4,-10.9)	<.001	-9.3 (-19.1,0.4)	.06
Alcohol consumption	1.0 (-12.5,14.6)	.87	3.7 (-7.6,15.0)	.51
Glucocorticoid use >3 months	-19.5 (-46.4,7.3)	.15	-19.9 (-56.1,16.1)	.27
Gait speed				
<0.70	8.7 (-6.0,23.4)	.24	2.9 (-9.5,15.5)	.64
0.70-0.84	6.2 (-8.7,21.1)	.41	4.3 (-8.9,17.5)	.52
0.85-1.01	5.3 (-8.5,19.2)	.45	12.9 (0.9,24.9)	.03
>1.01	0.0 (ref)		0.0 (ref)	
p for trend	.66		.10	
Five times sit to stand test completion time				
<7.2	-0.5 (-16.9,15.8)	.94	4.4 (-9.9,18.8)	.54
7.2-8.9	-6.4 (-21.2,8.3)	.39	4.4 (-8.1,17.1)	.48
9.0-10.8	-5.1 (-20.4,10.2)	.51	3.6 (-9.2,16.4)	.57
>10.8	0.0 (ref)		0.0 (ref)	
p for trend	.77		.79	
Sharpened Romberg test positive	-4.0 (-19.5,11.3)	.60	-16.6 (-28.9,-4.3)	.008
Rural residents	-19.5 (-29.7,-9.3)	<.001	-1.4 (-9.9,7.1)	.74

eTable 8. Linear regression analysis of risk factors associated with BMD in Chinese women aged 40 years or older

	L1-L4 (N=10022)		Total hip (N=10029)	
	**Regression coefficient (95% CI)**	***p* value**	**Regression coefficient (95% CI)**	***p* value**
Age				
40-49	0.0 (ref)		0.0 (ref)	
50-59	-117.5 (-128.8,-106.2)	<.001	-70.0 (-80.1,-59.9)	<.001
60-69	-210.0 (-221.7,-198.4)	<.001	-146.1 (-157.0,-135.3)	<.001
70-79	-241.0 (-257.9,-224.1)	<.001	-212.4 (-226.5,-198.3)	<.001
≥80	-265.6 (-300.9,-230.4)	<.001	-241.2 (-271.6,-210.7)	<.001
p for trend	<.001		<.001	
Body-mass index (kg/m^2)				
<18·5 (underweight)	-100.1 (-128.7,-71.5)	<.001	-98.9 (-119.4,-78.5)	<.001
18·5–23·9 (normal weight)	0.0 (ref)		0.0 (ref)	
≥24·0 (overweight and obese)	68.0 (58.7,77.3)	<.001	82.6 (74.4,90.9)	<.001
p for trend	<.001		<.001	
Parent Fractured Hip	-9.1 (-30.5,12.3)	.40	-17.0 (-33.4,-0.7)	.04
Ever-smoker	-6.2 (-36.5,24.0)	.68	-6.8 (-35.1,21.5)	.63
Alcohol consumption	-27.3 (-61.3,6.6)	.11	-3.7 (-23.9,16.5)	.71
Glucocorticoid use >3 months	-2.5 (-26.0,20.9)	.83	-19.8 (-44.6,4.9)	.11
Gait speed				
<0.70	1.5 (-12.0,15.1)	.82	-3.8 (-14.9,7.2)	.17
0.70-0.84	-0.5 (-13.2,12.2)	.93	4.2 (-6.1,14.6)	.42
0.85-1.01	-9.7 (-23.5,4.0)	.16	-8.6 (-21.0,3.8)	.49
>1.01	0.0 (ref)		0.0 (ref)	
p for trend	.81		.79	
Five times sit to stand test completion time				
<7.2	-13.8 (-26.6,-0.9)	.04	-9.8 (-20.7,1.0)	.08
7.2-8.9	-16.3 (-29.2,-3.4)	.01	-2.1 (-13.1,8.7)	.69
9.0-10.8	-6.8 (-20.4,6.7)	.32	3.9 (-8.6,16.5)	.54
>10.8	0.0 (ref)		0.0 (ref)	
p for trend	.003		.02	
Sharpened Romberg test positive	-17.3 (-31.4,-3.2)	.02	-19.9 (-34.5,-5.2)	.008
Rural residents	-27.4 (-35.9,-18.8)	<.001	-17.7 (-24.8,-10.7)	<.001

eTable 9. Multiply-adjusted odds ratios for vertebral fracture of grade 2 or above, and clinical fracture associated with risk factors in Chinese men aged 40 years or older

	Vertebral fracture of grade 2 or above (N=3556)		Clinical fracture in the past 5 years (N=7301)	
	**OR (95% CI)**	**p value**	**OR (95% CI)**	**p value**
Age (years)				
40-49	1.00 (ref)		1.00 (ref)	
50-59	1.38 (0.34 to 5.58)	.65	0.82 (0.49 to 1.36)	.44
60-69	2.00 (0.50 to 8.05)	.33	0.68 (0.39 to 1.19)	.18
70-79	2.68 (0.2 to 11.70)	.19	0.63 (0.32 to 1.25)	.18
≥80	3.29 (0.56 to 19.8)	.19	0.65 (0.17 to 2.45)	.52
p for trend		.10		.74
Femoral neck BMD (each SD decrement)	2.53 (1.68 to 3.81)	<.001	1.34 (1.05 to 1.70)	.02
Body-mass index (kg/m2)				
<18.5	0.33 (0.09 to 1.13)	.08	0.52 (0.17 to 1.57)	.24
18.5-23.9	1.00		1.00	
≥24	1.24 (0.73 to 2.11)	.43	1.20 (0.81 to 1.77)	.35
p for trend		.78		.31
Parent Fractured Hip	0.47 (0.13 to 1.63)	.23	1.02 (0.50 to 2.06)	.95
Ever-smoker	0.51 (0.26 to 0.99)	.05	1.10 (0.67 to 1.80)	.70
Alcohol consumption	1.80 (0.83 to 3.91)	.14	1.73 (1.08 to 2.78)	.02
Glucocorticoid use >3 months	1.54 (0.33 to 7.24)	.58	4.73 (1.14 to 19.50)	.03
Gait speed (m/s)				
<0.70	1.24 (0.44 to 3.48)	.68	0.91 (0.53 to 1.54)	.73
0.70-0.84	1.51 (0.52 to 4.35)	.45	1.38 (0.82 to 2.33)	.22
0.85-1.01	1.33 (0.48 to 3.73)	.58	0.90 (0.54 to 1.19)	.68
>1.01	1.00 (ref)		1.00 (ref)	
p for trend		.57		.27
Five times sit to stand test completion time (s)				
<7.2	1.00 (ref)		1.00 (ref)	
7.2-8.9	1.79 (0.70 to 4.59)	.22	1.05 (0.61 to 1.80)	.85
9.0-10.8	2.47 (0.89 to 6.84)	.08	1.24 (0.73 to 2.12)	.42
>10.8	3.18 (0.989 to 10.24)	.05	1.75 (0.97 to 3.16)	.06
p for trend		.09		.25
Sharpened Romberg test positive	1.73 (0.99 to 3.05)	.06	1.08 (0.64 to 1.82)	.75
Rural resident	1.71 (0.94 to 3.11)	.08	1.34 (0.92 to 1.96)	.12

eTable 10. Multiply-adjusted odds ratios for vertebral fracture of grade 2 or above, and clinical fracture associated with risk factors in Chinese women aged 40 years or older

	Vertebral fracture of grade 2 or above (N=4801)		Clinical fracture in the past 5 years (N=9998)	
	OR (95% CI)	p value	OR (95% CI)	p value
Age (years)				
40-49	1.00 (ref)		1.00 (ref)	
50-59	3.32 (1.21 to 9.11)	.02	1.92 (1.25 to 2.97)	.003
60-69	6.30 (2.30 to 17.23)	<.001	1.98 (1.22 to 3.23)	.006
70-79	12.35 (4.20 to 36.40)	<.001	1.33 (0.73 to 2.42)	.33
≥80	12.68 (3.71 to 43.33)	<.001	0.89 (0.31 to 2.55)	.83
p for trend		<.001		.25
Femoral neck BMD (each SD decrement)	2.17 (1.66 to 2.83)	<.001	1.26 (1.04 to 1.53)	.01
Body-mass index (kg/m2)				
<18.5	0.31 (0.10 to 0.98)	.05	0.60 (0.23 to 1.58)	.30
18.5-23.9	1.00		1.00	
≥24	1.14 (0.66 to 1.97)	.65	1.34 (1.00 to 1.80)	.05
p for trend		.85		.07
Parent Fractured Hip	0.46 (0.19 to 1.09)	.08	1.45 (0.88 to 2.39)	.13
Ever-smoker	0.66 (0.17 to 2.60)	.55	1.08 (0.50 to 2.31)	.83
Alcohol consumption	1.23 (0.41 to 3.73)	.71	1.22 (1.55 to 2.67)	.61
Glucocorticoid use >3 months	1.21 (0.31 to 4.82)	.78	1.87 (0.92 to 3.79)	.08
Gait speed (m/s)				
<0.70	2.26 (0.99 to 5.13)	.05	1.28 (0.83 to 1.98)	.25
0.70-0.84	2.23 (0.99 to 5.02)	.05	0.91 (0.58 to 1.42)	.68
0.85-1.01	2.28 (1.09 to 4.77)	.03	1.25 (0.81 to 1.93)	.30
>1.01	1.00 (ref)		1.00 (ref)	
p for trend		.27		.13
Five times sit to stand test completion time (s)				
<7.2	1.00 (ref)		1.00 (ref)	
7.2-8.9	3.03 (1.05 to 8.76)	.04	1.39 (0.87 to 2.23)	.16
9.0-10.8	1.84 (0.61 to 5.60)	.28	1.61 (0.99 to 2.61)	.05
>10.8	2.18 (0.79 to 6.06)	.13	2.12 (1.32 to 3.42)	.002
p for trend		.79		.02
Sharpened Romberg test positive	1.53 (0.92 to 2.56)	.10	1.23 (0.89 to 1.71)	.19
Rural resident	0.95 (0.57 to 1.58)	.83	0.91 (0.70 to 1.17)	.47

eFigure 1. Geographic distribution of 11 selected provinces/municipalities in the study.

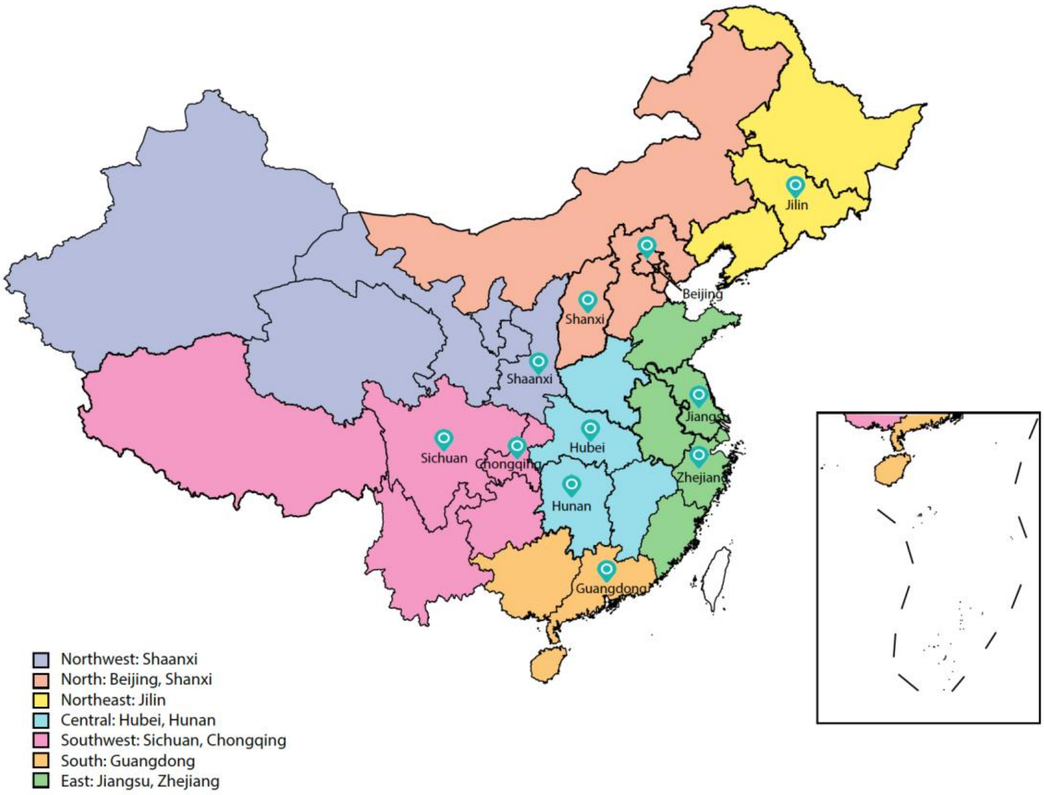

eFigure 2. Smoothed curve of mean bone mineral density (BMD) by age in Chinese population aged 20 years and over. A, Smoothed curve of mean BMD by age in men, showing that the peak BMD in men reached at 20-29 years at all measured sites; B, smoothed curve of mean BMD by age in women, showing that the peak BMD in women reached at 30-39 years at lumbar spine and 20-29 years at femoral neck and total hip.

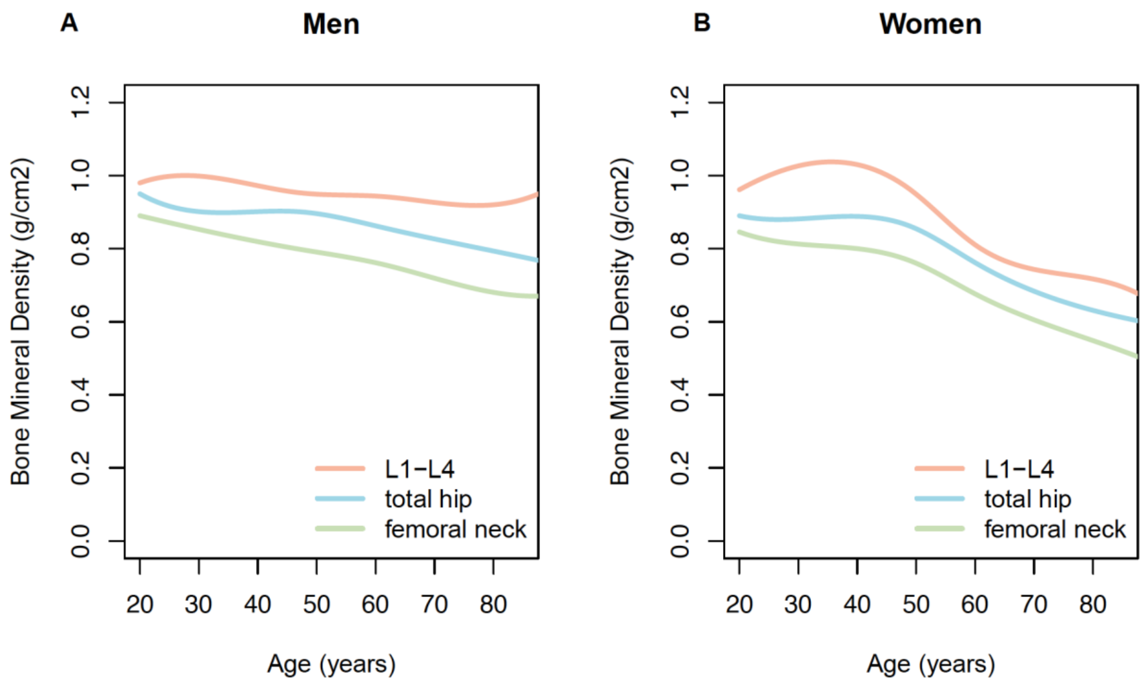

eMethods.

1. The process of sample size calculation in the study.

In the study design, we enrolled people aged 20-39 years for establishing the peak BMD, and people aged 40 years or older for studying the prevalence of osteoporosis. The sample size for each study aim was calculated as follows:

(1) Estimating the sample size for establishing the peak BMD (using people aged 20 - 39 years).

The following formula was used: sample size N = $(\mu_\alpha \sigma/\delta)^2$. α = 0.05; total sample deviation σ were 0.090 – 0.196 g/cm^2 (based on the current knowledge of the standard deviation of the BMD in people aged 20 – 39 years), and we used σ = 0.196 g/cm^2 for this study; the permissible error δ = 25%×σ= 25%×0.09=0.0225. Based on the parameters above, the sample size N was 279 for each stratum. There were two strata of genders (male and female), two strata of ages (20-29 years and 30-39 years), and two strata of urban district-rural county, so the total sample size N = 2232. We assumed the response rate to be 80%, so the total sample size in minimum was 2790. To achieve enough sample size, we sampled 64 people for each urban district or rural county. There were 44 urban districts/rural counties, so the total sample size was 2816.

(2) Estimating the sample size for studying the prevalence of osteoporosis (using people aged 40 years or older)

The following formula was used : sample size N = *deff* $\frac{\mu_\alpha^2\, p(1-p)}{d^2}$. α= 0.05 (two-tailed); the prevalence of osteoporosis *p* = 13.2% (based on literature); the study efficiency *deff* = 3; the relative error *r* = 15%. Based on the parameters above, N was calculated as 3369 for each stratum. There were two strata of genders (male and female), and two strata of urban district-rural county, so the total sample size N = 13476 in total. We assumed the response rate to be 80%, and the sample size in minimum was estimated as 16845. To achieve enough sample size, we sampled 400 for each urban district or rural county. There were 44 urban districts/rural counties, so the total sample size was 17600.

2. The inclusion and exclusion criteria of participants.

We only included permanent residents living in sampling sites for six months or longer during the last 12 months into the study; and we excluded participants if: 1) living in work sheds, military camps, dormitories, or nursing homes; or 2) having cognitive disorder or communication disorder; or 3) being high paraplegia; or 4) being pregnant; or 5) unable to lie on the examination bed for five minutes; or 6) having spinal deformity or having metal implantation in the spine.

3. The definition of risk factors in the questionnaire.

We defined alcohol consumption as drinking alcohol at least once a week, and glucocorticoid use as having continuously used glucocorticoid for over three months. We measured height and weight following standard protocols, and gait speed as time to walk

2.5 meters at normal pace, repeating and taking the average of two tests. The Five Times Sit to Stand Test measured time sitting with back against the chair and standing up as quickly as possible for five times without support of arms. The Sharpened Romberg test asked participants to stand with feet tandem over thirty seconds (for aged ⩾ 70 years) or over sixty seconds (for aged <70 years).

4. The protocol of cross calibration among different DXA scanners.

A lumbar phantom provided by the manufacturer was measured daily at each site for longitudinal quality control following the classical Shewhart control chart rule[1]. Coefficient of variance should be kept between -1.5% to +1.5%. For cross calibration among different DXA scanners, we scanned European spine phantoms (ESP) in each site with the same standardized scan protocol used for scanning participants in the study.[2,3] We used linear regression to adjust the scanned BMD values by true values of QRM-ESP (0.5, 1.0, and 1.5 g/m2). We established a linear regression equation for each DXA scanner to standardize the BMD values[4-7]. The calculation of peak BMD and prevalence of osteoporosis were all based on the adjusted BMD. Two senior radiologists (WY and QL) evaluated all DXA printouts according to standard operating procedure.

Reference:

1. Lu Y, Mathur AK, Blunt BA, et al. Dual X-ray absorptiometry quality control: comparison of visual examination and process-control charts. J Bone Miner Res. 1996;11(5):626-637.
2. Dequeker J, Reeve J, Pearson J, et al. Multicentre European COMAC-BME study on the standardisation of bone densitometry procedures. Technol Health Care. 1993;1(2):127-131.
3. Pearson J, Ruegsegger P, Dequeker J, et al. European semi-anthropomorphic phantom for the cross-calibration of peripheral bone densitometers: assessment of precision accuracy and stability. Bone Miner. 1994;27(2):109-120.
4. Dequeker J, Reeve J, Pearson J, et al. Multicentre European COMAC-BME study on the standardisation of bone densitometry procedures. Technol Health Care. 1993;1(2):127-131.
5. Pearson J, Ruegsegger P, Dequeker J, et al. European semi-anthropomorphic phantom for the cross-calibration of peripheral bone densitometers: assessment of precision accuracy and stability. Bone Miner. 1994;27(2):109-120.
6. Hans D, Downs RW, Jr., Duboeuf F, et al. Skeletal sites for osteoporosis diagnosis: the 2005 ISCD Official Positions. J Clin Densitom. 2006;9(1):15-21.
7. Kanis JA, Kanis JA. Assessment of fracture risk and its application to screening for postmenopausal osteoporosis: Synopsis of a WHO report. Osteoporosis International. 1994;4(6):368-381.
